# Comprehensive dissection of dispensable genomic regions in *Streptomyces* based on comparative analysis approach

**DOI:** 10.1186/s12934-020-01359-4

**Published:** 2020-05-06

**Authors:** Qing-Ting Bu, Yue-Ping Li, Huang Xie, Jue Wang, Zi-Yue Li, Xin-Ai Chen, Xu-Ming Mao, Yong-Quan Li

**Affiliations:** 1 Institute of Pharmaceutical Biotechnology and Research Center for Clinical Pharmacy of First Affiliated Hospital, Zhejiang University School of Medicine, Hangzhou, 310058 China; 2 Zhejiang Provincial Key Laboratory for Microbial Biochemistry and Metabolic Engineering, Hangzhou, 310058 China

**Keywords:** Genome reduction, Chassis, Synthetic lethality, Multi-omics, Essential genes, *Streptomyces*

## Abstract

**Background:**

Large-scale genome reduction has been performed to significantly improve the performance of microbial chassis. Identification of the essential or dispensable genes is pivotal for genome reduction to avoid synthetic lethality. Here, taking *Streptomyces* as an example, we developed a combinatorial strategy for systematic identification of large and dispensable genomic regions in *Streptomyces* based on multi-omics approaches.

**Results:**

Phylogenetic tree analysis revealed that the model strains including *S. coelicolor* A3(2), *S. albus* J1074 and *S. avermitilis* MA-4680 were preferred reference for comparative analysis of candidate genomes. Multiple genome alignment suggested that the *Streptomyces* genomes embodied highly conserved core region and variable sub-telomeric regions, and may present symmetric or asymmetric structure. Pan-genome and functional genome analyses showed that most conserved genes responsible for the fundamental functions of cell viability were concentrated in the core region and the vast majority of abundant genes were dispersed in the sub-telomeric regions. These results suggested that large-scale deletion can be performed in sub-telomeric regions to greatly streamline the *Streptomyces* genomes for developing versatile chassis.

**Conclusions:**

The integrative approach of comparative genomics, functional genomics and pan-genomics can not only be applied to perform a multi-tiered dissection for *Streptomyces* genomes, but also work as a universal method for systematic analysis of removable regions in other microbial hosts in order to generate more miscellaneous and versatile chassis with minimized genome for drug discovery.

## Background

Large-scale genome reduction has been performed to streamline microbial genomes in order to decrease metabolic burden as far as possible, and to further develop simplified and versatile chassis for producing valuable amino acids, peptides, fuels or drugs [1–4]. Identification of the essential or redundant genes is pivotal for genome reduction to avoid synthetic lethality [5]. Comparative genomic approaches and large-scale gene inactivation technologies have been developed to investigate essential or dispensable genes [6]. On the one hand, based on the hypothesis that essential genes are highly conserved in the process of evolution, multiple genome alignment can be performed to predict essential genes rapidly [7]. On the other hand, gene inactivation methods, also called experimental approaches, like global transposon mutagenesis, antisense RNA technique, single-gene knockout and allelic replacement mutagenesis have been used to determine the essentiality of genes from the perspective of functions directly [8, 9]. However, gene inactivation methods are time-consuming and labor-intensive which are not suitable for large-scale genome deletion, especially in larger genomes. Nonetheless, essential genes identified by experimental approaches are gathered to develop the Database of Essential Genes (DEG) in order to facilitate the prediction of essential genes in other sequenced genomes [10]. Currently, comparative genomics was the main approach to analyze non-essential and removable genomic regions, which mainly include biosynthesis gene clusters (BGCs), mobile genetic elements (MGEs), genome islands (GIs), insertion sequences (ISs) or other elements from horizontal gene transfer (HGT), on a large scale. For example, 15 putative and dispensable secondary metabolite BGCs were located in the genome of the *Streptomyces albus* J1074 by antiSMASH analysis. By removing the 15 BGCs spanning 503 kb in sequence, a cluster-free mutant *S. albus* Del14 was constructed and the mutant Del14 has successfully served as chassis for improving the production of microbial drugs and activating cryptic gene clusters [11]. Based on the *Escherichia coli* Data Banks, the IS-free *E. coli* MS56 was constructed by sequentially deleting all ISs and K-islands, and the mutant MS56 harboring 23% genomic deletion showed enhancing genomic stability and recombinant protein production [12]. By comparing the genomes of *E. coli* MG1655 and EDL933, 12 K-islands were identified and deleted to construct a genome-reduced host MDS12 [13]. Another example is the construction of genome-minimized *Streptomyces* hosts *Streptomyces avermitilis* SUKAs with large-deletion. Comparative analysis of *S. avermitilis*, *Streptomyces coelicolor* A3(2) and *Streptomyces griseus* revealed two sub-telomeric regions at the left and right chromosomal ends of *S. avermitilis*. A > 1.4-Mb segment from the left sub-telomeric region was deleted directly by Cre/*loxP* recombinant system and further endogenous gene clusters were removed one by one to construct a series of genome-streamlined *S. avermitilis* mutants SUKAs which have been widely used to efficiently express a variety of heterologous gene clusters [14]. Besides, based on comparative genomic analysis, genome-reduction has been performed in various microbial cells like *E. coli*, *Bacillus subtilis*, *Pseudomonas putida*, *Cyanobacterium*, *Aspergillus nidulans*, *Schizosaccharomyce pombe* and *Streptomyces* [14–20]. As expected, many genome-reduced cells exhibited emergent and excellent performances like higher transformation efficiency, shortened growth cycle, increased genomic stability, enriched intracellular energy and reducing power, and enhanced production of heterologous proteins or secondary metabolites [21]. The favorable effect of genome reduction encourages us to develop more systematic approaches to determine the large dispensable and removable regions in microbial genomes. Currently, multiple genome alignment is the main method to analyze the non-essential genomic regions, however, this method is one-sided and risky which may result in lethality when the putative regions are knocked out. Therefore, it is an urgent need to develop more systematic strategies for dissecting dispensable genomic regions.

All the time, *Streptomyces* spp. are the main antibiotic-producers which can biosynthesize antibiotics, antitumor agents, immunesuppressor, antioxidants, antihypertensive and hypoglycemic drugs widely used in clinical practice [22, 23]. Generally, *Streptomyces* species can synthesize a variety of precursors and elements, and harbor 20–40 gene clusters on average in their genomes which mightily indicates their robust capabilities of primary and secondary metabolism. Therefore, *Streptomyces* species are the preferred chassis for heterologous activation or overproduction of valuable microbial or plant drugs [24]. However, during long-term evolution, a large number of non-essential elements like mobile genetic elements (MGEs), genomic islands (GIs) and biosynthetic gene clusters (BGCs) are integrated into the genomes of *Streptomyces* by phage infection or horizontal gene transfer. The MGE like insertion sequences (ISs), transposons can randomly move and insert into other loci of genomes, and may result in genome rearrangement, gene inactivation or deletion, which affect genomic stability [25]. The endogenous BGCs not only consume plenty of energy, reducing power and precursor, but also produce lots of non-target products which interferes isolation and purification of target products [26]. Therefore, to refactor the genomes by large-scale deletion of non-essential elements will greatly improve the performance of *Streptomyces* hosts for drug discovery and high-yield.

**Fig. 1:**
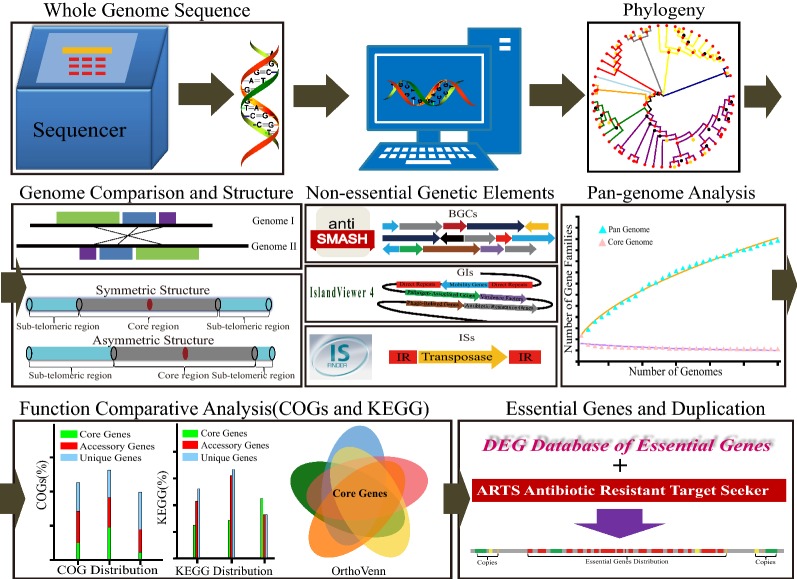
Schematic representation of the genomic analysis workflow based on a multi-omics approach

Here, taking *Streptomyces* as an example, we developed an integrative strategy for systematically analyzing large and dispensable genomic regions in *Streptomyces* based on multi-omics approaches. The analytical hierarchy process was completed according to the flowchart in Fig. 1. This strategy can not only avoid the lethality of large-scale deletion for convenience of rational construction of genome-streamlined and hyper-performing *Streptomyces* hosts, but also can be widely used in other microbial cells to systematically analyze abundant genes for generating more miscellaneous and versatile chassis with minimized genomes.

## Results

### Phylogenetic tree analysis

**Fig. 2:**
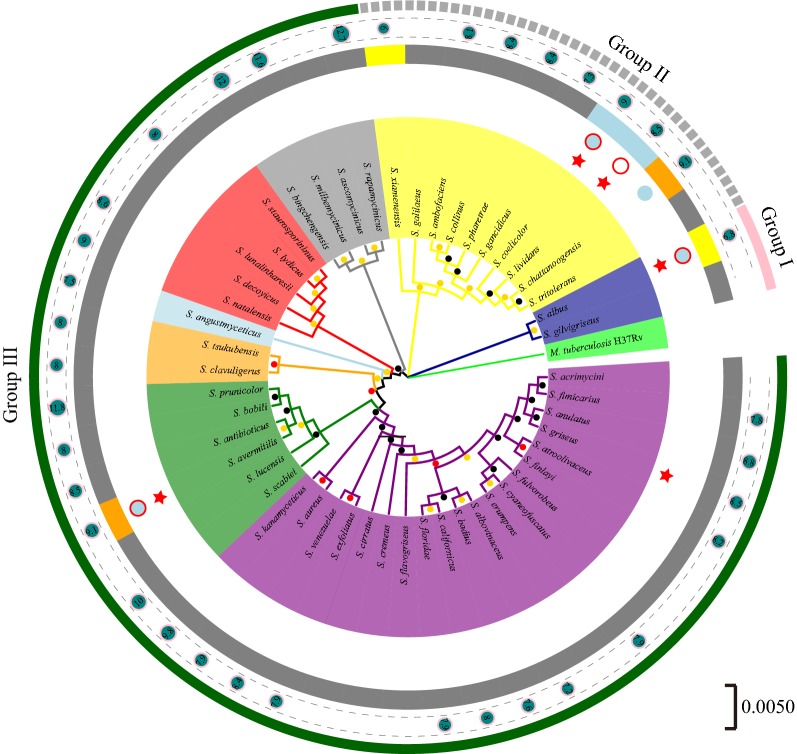
Phylogenetic tree of the selected 50 *Streptomyces* strains. Circle 1 shows the taxonomically distinct groups: Group I, II and III. Circle 2 shows the whole genome of part of *Streptomyces* and the number in the dark green circles displays the genome size. Circle 3 displays the *Streptomyces* with naturally minimized genomes (yellow strap) and with large-scale deletion (orange strap). Circle 4 displays the genome-minimized chassis (light-blue circle) and widely used hosts (red ring). Circle 5 displays the representative model *Streptomyces* (red star). *Mycobacterium tuberculosis* H37Rv was used as outgroup

In theory, essential genes are more evolutionarily conserved than non-essential genes among different strains. However, non-essential genes may be well conserved between taxonomically closely related species which will make us overestimate the amount of essential genes. Therefore, in order to systematically predict the essential genes by computational approaches, we need to firstly investigate the evolutionary relationship between different strains by phylogenetic tree analysis. Here, 50 16S ribosomal DNA (rDNA) sequences of *Streptomyces* genomes were selected to construct phylogenetic tree using neighbor-joining algorithm in the MEGA software [27]. The genome sequences were freely available from NCBI. The phylogenetic tree was further visualized and edited by the EvolView online tool [28]. We can see that 50 strains were roughly divided into three taxonomically distinct groups: Group I, II and III (Fig. 2). Several model representatives of *Streptomyces*, *S. albus* J1074, *S. lividan* TK24 and *S. coelicolor* A3(2), *S. avermitilis* MA-4680 and *S. griseus* belonged to Group I, II and III, respectively. As is well-known, *S. albus* J1074 and *S. xiamenensis* 318 were two naturally genome-minimized strains harboring 6.84 Mb- and 5.96 Mb-size genomes, respectively. Besides, *S. coelicolor* A3(2), *S. albus* J1074, and *S. avermitilis* MA-4680 are three well-studied models and have been genetically engineered to generate genome-streamlined mutants which work as versatile hosts for heterologous expression of value-added natural products. Therefore, the genomes of *S. coelicolor* A3(2), *S. albus* J1074 and *S. avermitilis* MA-4680 are preferred references for comparative analysis of target genomes.

### Comparative analysis of *Streptomyces* genomes

**Fig. 3:**
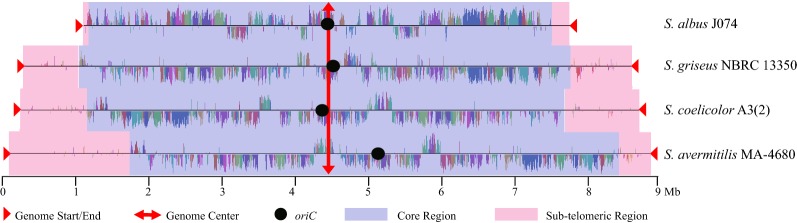
Multiple genome alignment and genome structure. Comparative analysis of four taxonomically distinct *Streptomyces* genomes, *S. avermitilis* MA‑4680, *S. coelicolor* A3(2), *S. albus* J074 and *S. griseus* NBRC 13350, revealed a conserved core region (lightblue strap) in which the majority of the genes are highly conserved with a high degree of synteny and two sub-telomeric regions (pink strap) located at the end of the chromosome. The red triangles display the genome start or end. The red arrow displays the location of genome center. The black circle shows the replication of origin (*oriC*)

During the process of evolution, genes responsible for fundamental functions like DNA replication, transcription, translation, primary metabolism, cell division should be well conserved. Based on the above phylogenetic tree analysis, we performed comparative genome analysis of several taxonomically distinct *Streptomyces* genomes which revealed highly conserved core region and variable sub-telomeric regions (Fig. 3). In Fig. 3, the distribution of conserved genes can also be seen visually with similarity plot. We found that the conservation of genes in core region was very high and some functionally related genes were clustered like carbon and nitrogen metabolism which may be conducive to the interaction of enzymes. We observed that in the naturally genome-minimized *S. albus* J1074, only two less conserved regions (0.2 Mb and 0.3 Mb) were located at the end of its genome, and there were no apparent and large sub-telomeric regions, and most genes are highly conserved. However, two distinct and less conservative regions were observed in the genomes of *S. coelicolor* A3(2), *S. griseus* and *S. avermitilis* MA-4680. And the size of sub-telomeric regions was approximately same (1 Mb) in the genomes of *S. coelicolor* A3(2) and *S. griseus*, but was unequal (1.5 Mb and 0.5 Mb) in the genome of *S. avermitilis* MA-4680. Similar asymmetric distribution was also noticed in the genome of *S. chattanoogensis* L10 according to our previous study [29]. We supposed that the asymmetry of two sub-telomeric regions in *S. avermitilis* MA-4680 and *S. chattanoogensis* L10 may attribute to the deviation (0.7 Mb and 0.77 Mb) of *oriC* to the center of chromosome. In order to make it more convincing, we performed multiple genome alignment with *Streptomyces bingchenggensis* BCW-1 in which the *oriC* had a 0.64 Mb deviation to the center (Additional file 1). The result suggested that two sub-telomeric regions were also asymmetric, 2.76 Mb and 1.41 Mb, respectively, at the end of the chromosome. In the meantime, we noticed that the core region was distributed around the *oriC* as its axis of symmetry. We also observed that most conserved genes were concentrated in the core region, and the symmetric pattern of core region to *oriC* may be conducive to the prior expression of essential genes or avert frequent rearrangement, deletion and mutation at sub-telomeric region which maybe an adaptive protection mechanism of cells. Therefore, we guessed that a large number of non-essential genes may be mainly distributed in the sub-telomeric regions. We needed to further verify our conjecture by analyzing well-known non-essential elements.

### Genomic analysis of putative non-essential elements

The majority of secondary metabolite biosynthesis gene clusters (BGCs), genomic islands (GIs) and insertion sequences (ISs) mainly from horizontal gene transfer or bacteriophage infection, belong to well-known non-essential elements. The antiSMASH [30], IslandViewer [31] and ISsaga [32] softwares were used to analyze the BGCs, GIs and ISs, respectively. The result was shown in Table 1.

**Table 1:** Genomic characters of four representative model *Streptomyces*

*Streptomyces*	*S. albus* J1074	*S. coelicolor* A3(2)	*S. griseus* NBRC 13350	*S. avermitilis* MA-4680
NCBI number	NC_020990	NC_003888.1	NC_010572.1	NC_003155.1
Genome size	6841649 bp	8667507 bp	8545929 bp	9025608 bp
Chromosome center	3420825 bp	4333754 bp	4272965 bp	4512804 bp
*oriC* location	3419111 bp-3420244 bp	4269844 bp-4270777 bp	4324371 bp-4325375 bp	5287934 bp-5289023 bp
*oriC* to center deviation	1147 bp(left)	63443 bp(left)	51909 bp(right)	775675 bp(right)
Core region	461493 bp-6274525 bp	1109358 bp-7552504 bp	1100483 bp-7490380 bp	1735832 bp-8475739 bp
BGCs count	22	30	38	36
BGCs size	971608 bp	994310 bp	1631915 bp	1510537 bp
BGCs distribution in sub-telomeric regions	54%	45%	52%	40%
GIs count	44	94	99	21
GIs size	411976 bp	1092300 bp	1270295 bp	594367 bp
GIs distribution in sub-telomeric regions	9.9%	46.7%	67.5%	77.5%
ISs count	53	107	75	173
ISs size	49359 bp	99533 bp	96856 bp	229749 bp
ISs distribution in sub-telomeric regions	16.1%	32.2%	45.4%	55.4%

We can see that the BGCs accounted for a great proportion in the whole genomes (14% for *S. albus* genome, 11% for *S. coelicolor* genome, 19% for *S. griseus* and 17% for *S. avermilitis* genome) and lots of BGCs were distributed in sub-telomeric regions (54% in *S. albus* genome, 45% in *S. coelicolor*, 52% in *S. griseus*, and 40% in *S. avermilitis* genome). Although the GIs and ISs took up a smaller proportion, 30-70% of them were also concentrated in the sub-telomeric regions of each genome except *S. albus* genome. The concentrated distribution of non-essential elements suggested the plasticity and editability of *Streptomyces* genomes which impelled us to engineer and refactor the genomes for improving their performances. As previously reported, in certain *Streptomyces* species like *S. albus* BK3-25 [33] and *S. ambofaciens* [17], the arms were unstable and can undergo spontaneous homologous recombination, deletions, or duplication which may be due to the terminal inverted repeats sequences (TIRs) or the mobile genetic elements like transposons, insert sequences or genome islands. Many studies have proved that deletion of mobile genetic elements can improve the stability of genomes, for example, in *E. coli* [12]. In our previous study, our results also indicated that deletion of insertion sequences (ISs) in industrial *S. chattanoogensis* may decrease IS-mediated random mutagenesis and increase its genetic stability [29]. Here, we also noticed that the number of mobile genetic elements in industrial *Streptomyces* genomes was more than that in model ones which maybe the reason why many industrial *Streptomyces* were more unstable. Although large-scale deletion of sub-telomeric regions had been performed to generate genome-minimized mutant in *S. avermilitis*, this method may bring about risk of lethality because some essential genes may be dispersed in sub-telomeric regions. Therefore, it is very necessary to further identify essential genes and their distributions.

### Pan-genomic analysis

**Fig. 4:**
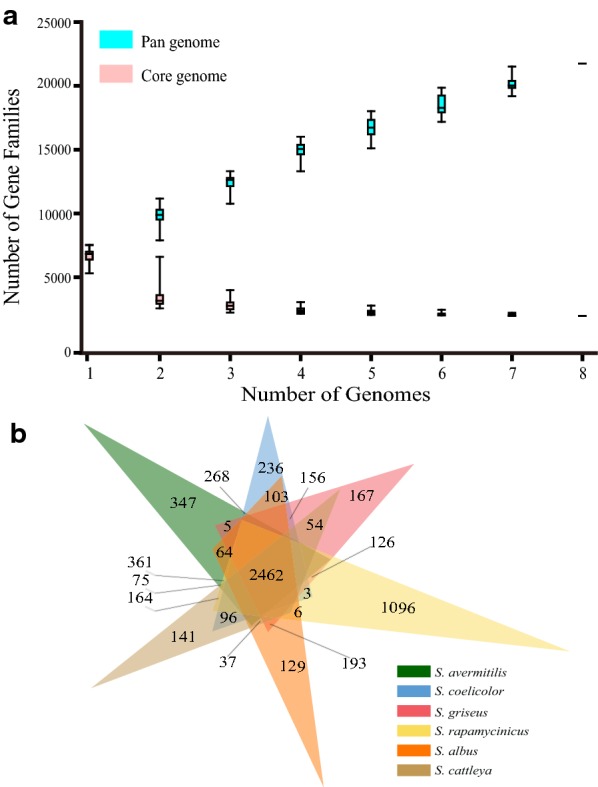
Pan-genome analysis of divergent *Streptomyces* genomes. **a** The number of core genes gradually decreased along with the increase of the number of given genomes. The lightblue boxplots display the number of gene families of pan genome. The pink boxplots show the number of gene families of core genome. **b** Comparison of orthologous gene clusters by OrthoVenn

Pan-genome analysis can be performed to determine the core genes, accessory genes, and strain-specific (unique) genes in given genomes. According to the phylogenetic tree of 50 *Streptomyces* genomes, we selected 8 divergent species for the pan-genome analysis with BPGA [34]. We can see that the number of core genes gradually decreased along with the increase of the number of given genomes. Only about 2500 core genes were well conserved in each genome which may belong to putative essential genes (Fig. 4a). In order to further determine the essential genes and their functional categories, we performed comparison and annotation of orthologous gene clusters with protein sequences among multiple Streptomyces species by OrthoVenn [35]. The result indicated that about 2460 orthologous genes were shared by each genome which was consistent with the result of pan-genome analysis (Fig. 4b). The main functions in the co-orthologous genes were related to the basal metabolisms, suggesting that co-orthologous genes should be essential for basic cellular functions. The distribution of functional categories for the core, accessory genes and unique genes showed that the main functions in the core genes were linked to DNA replication, transcription and translation, energy production and conversion, coenzyme transport and metabolism, or primary metabolic processes.

### Comparative analysis of functional genomes

**Fig. 5:**
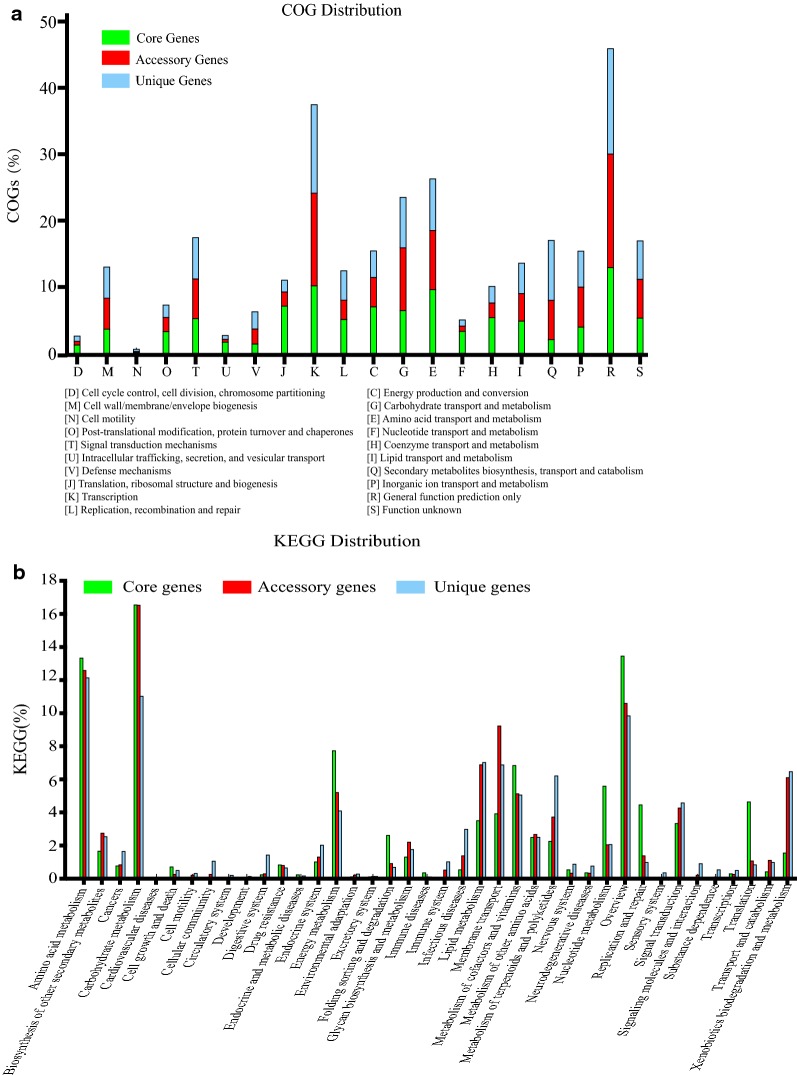
Analysis of COG and KEGG distributions, and functional categories of essential genes. **a** The COG distribution. **b** The KEGG distribution

Analysis of COG distribution suggested that the overall proportion of secondary metabolic process-associated functions among the accessory and unique genes was approximately 80%, compared with the core genes (Fig. 5a). The KEGG analysis revealed that the amino acid, carbohydrate, lipid and cofactors metabolisms accounted for a relatively large proportion both in the core, accessory and unique genes. In the categories of energy metabolism, protein folding sorting and degradation, cofactors and vitamins metabolisms, nucleotide metabolism, replication and repair, or translation, the core genes took up a greater proportion than the accessory and unique genes (Fig. 5b). Besides, we also noticed that there existed two or more copies of some essential genes with function duplication. Our previous study suggested that synchronous deletion of multiple copies of essential genes may lead to cell death and this phenomenon was also called synthetic lethality [29]. Therefore, to investigate the distribution of essential genes and their duplications was also vital for us to construct genome-minimized hosts by large-scale deletion. The ARTS (Antibiotic Resistant Target Seeker) has been developed to analyze BGCs, core (essential) genes and their duplications, and known resistance models [36]. Four *Streptomyces* genomes were submitted to ARTS to analyze well-known essential genes and their duplications. The well-known essential genes were identified using ARTS with its comparative pipeline and TIGRfam Equivologs. Generally, these genes belonged to core genes which were responsible for fundamental cellular functions like DNA replication, transcription, translation, primary metabolic pathways and cell architecture. These genes were generated by comparative genomic analysis and the reference genes in ARTS were from published literatures. The result was shown in Table 2.

**Table 2:** Well-known essential genes and their duplications of four model *Streptomyces*

*Streptomyces*	Well-known essential genes	Duplications
*S. albus* J1074	390	32
*S. coelicolor* A3(2)	397	47
*S. griseus* NBRC 13350	387	30
*S. avermitilis* MA-4680	390	45

**Fig. 6:**
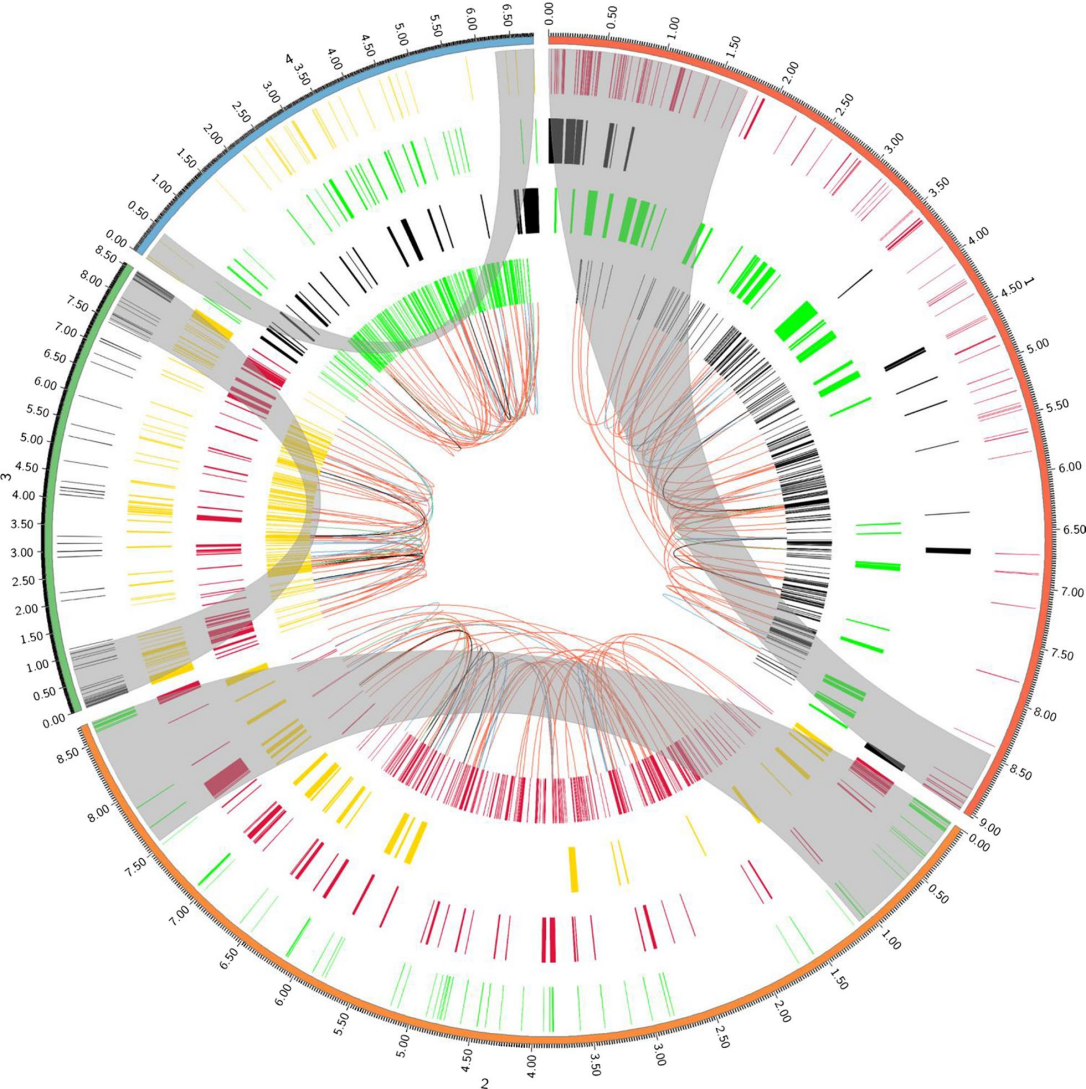
The circular map of four representative model *Streptomyces* genomes and the visualized distribution of non-essential regions and essential genes. The outmost circle (Circle 1) with tick mark shows the four genomes, **1***S. avermitilis* MA-4680, **2***S. coelicolor* A3(2), **3***S. griseus* NBRC 13350 and **4***S. albus* J1074. Circle 2 shows the location of ISs. Circle 3 shows the distribution of GIs. Cirlce 4 shows the location of BGCs. Circle 5 shows the distribution of well-characterized essential genes. Circle 6 shows the links between essential genes and their duplications. The gray stripes show the sub-telomeric regions

The result suggested that only about 390 well-known essential genes were identified in each genome, in which about 30–50 genes contained at least two copies. Analysis of the distribution of essential genes and their copies suggested that most of well-known essential genes were dispersed in core genomic region (96% in *S. avermilitis*, 96% in *S. griseus*, 94% in *S. coelicolor* and 97% in *S. albus*) and some copies of several essential genes were also located at the sub-telomeric regions. To further analyze the locations of these copies, we found that different essential genes and their copies did not appear in two sub-telomeric regions simultaneously which indicated that large-scale deletion of sub-telomeric regions did not result in synthetic lethality (Fig. 6). The above results by computational approaches were consistent with previous results by experiments. For example, Zhou’s study has proved that a 0.9-Mb segment at the left sub-telomeric region of *S. coelicolor* genome was deletable [37]. Mamoru Komatsu et al. have deleted about 1.4-Mb segment from the left sub-telomeric region of the *S. avermitilis* genome [14]. Our previous research also showed that two large segments (1.3-Mb and 0.7-Mb) at the left and right sub-telomeric regions of *S. chattanoogensis* L10 can be deleted separately. Besides, the two large segments in *S. chattanoogensis* L10 genome cannot be removed at the same time because several essential genes and their copies were dispersed in the two target regions simultaneously [29]. Therefore, analysis of the distribution of essential genes and their duplications was also pivotal for identification of deletable regions.

## Discussion

All the time, the construction of efficient and versatile chassis is one of the main research contents of synthetic biology. Many studies have revealed that many unnecessary genes distributed in native genomes resulted in extreme metabolic burden or genome instability [21]. For example, lots of endogenous secondary metabolite biosynthesis gene clusters in microbial genomes may not only consume precursors, energy and reducing power, but also produce a number of by-products. The insertion sequences known as transposable elements can move from one locus to another one randomly which usually leads to genome instability [38]. Nowadays, two kinds of strategies have been developed to streamline native genomes for generating genome-reduced chassis: the top-down and bottom-up approaches [39]. The bottom-up method is to chemically synthesize functional genomes which only contain essential genes responsible for fundamental cellular functions. Currently, it still is a great challenge to synthesize a complete genome because of the complexity and sophistication of genomes. In contrast, the top-down method is to remove dispensable genes from native genomes to generate streamlined genomes, which is more feasible in practice than the bottom-up strategy. The top-down strategy has been used to construct a series of genome-reduced microbial hosts. For example, a 1.4-Mb segment is removed directly from *S. avermitilis* MA-4680 by Cre/*loxP* recombination system to generate the large-deletion mutant *S. avermitilis* SUKA3 [14]. 15 gene clusters spanning about 0.5 Mb are deleted orderly from *S. albus* J1074 to generate a cluster-free mutant *S. albus* Del14 [11]. A 1.3 Mb or 0.7 Mb non-essential regions can be removed separately in the *S. chattanoogensis* L10 genome [29]. A deletion of ~ 0.7 Mb is made in the *E. coli* MG1655 by removing ISs, K-islands and other segments [4]. A *B. subtilis* mutant MG1M harboring about 1 Mb deletion is created by serially deleting 17 unnecessary regions [16]. Genome reduction is also achieved in other microbial cells like *Pseudomonas putida* KT2440, *Aspergillus nidulans*, *Saccharomyces cerevisiae* and *Schizosaccharomyces pombe* [39]. The emergent properties of genome reduction in different microbial cells drive us to seek more efficient and precise computational approaches to analyze dispensable elements for creating genome-minimized and performance-excellent chassis.

As mentioned above, the first step to streamline genomes is to design the removable segments systematically. Comparative genomic analysis has been performed to identify non-essential elements in genomes like BGCs, ISs, GIs or less conservative regions. However, previous comparative genomic approaches are mainly based on the multiple alignments of nucleotide sequences among different genomes, which is hard to define the removable segments systematically. *Streptomyces* species as the main producers of natural products are ideal and preferred chassis for natural product discovery and overproduction. Here, taking the *Streptomyces* as an example, we combined comparative genomics, functional genomics and pan-genomics to perform a multi-tiered dissection for *Streptomyces* genomes and tried to establish a universal method for systematical analysis of removable regions. Our approach to identify non-essential genes was mainly focused on the functions of genes and to avoid synthetic lethality from the perspective of core genes. Here, we integrated the all-round analysis of unique gene, clustered genes, and key pathway genes by multi-omics to systematically investigate the genomic architecture to guide researchers for rational construction of genome-reduced chassis by large-scale deletion. In brief, firstly, we need to perform phylogenetic tree analysis to select taxonomically distinct species in order to avoid the overestimation of essential genes. Although the genomes of *S. coelicolor* A3(2), *S. albus* J1074 and *S. avermitilis* MA-4680 are preferred references for comparative analysis of target genomes, for different candidate genomes, a new phylogenetic analysis should be made to choose taxonomically distinct genomes with the target one. Secondly, comparative genomic analysis and location of *oriC* were performed to determine the genome structure and non-essential elements. Our results suggested that there exist a highly conserved core region (~ 6 Mb) around the *oriC* as its axis of symmetry and two sub-telomeric regions at the end of chromosomes. Different arms might have gone through rounds of deletion, insertion and/or duplication to achieve the balance of replication [18]. For asymmetric structure, large deletion in the longer arms may contribute to the balance of replication and enhance the genetic stability. Comparative genomic analysis indicates that many dispensable elements like BGCs, ISs and GIs are dispersed in the sub-telomeric regions. Finally, functional and pan-genomic analyses were carried out to investigate the functions and essentialities of predicted target regions. Functional genomic analysis shows that the majority of genes in the sub-telomeric regions are unnecessary and removable. Analysis of well-known essential genes and their duplication reveals that large-scale deletion can be performed in the sub-telomeric regions to simplify the *Streptomyces* genomes, which may greatly improve the performance of strains. The expansion of the genetic repertoire of an organism by gene duplication can aid adaptation. Expanding gene families can help maintain cell functionality during metabolic perturbation [19]. However, we think that the nutrition is rich in the laboratory environment and most of gene copies may be redundant which had been proved by experiment in our previous study. We also found that most of core essential genes were involved in primary metabolism and not in secondary metabolism which indicated the conservation and necessity of function of core essential genes. Besides, a part of non-essential elements distributed in the core region can also be deleted sequentially to make the genomes more simplified and more stable, and to construct a genome-minimized, BGC-free, GI-free and IS-free chassis. The above analysis was performed based on the complete genomes. In fact, these methods were also suitable for some not complete genomes. Although large deletion cannot be performed in not complete genomes, researchers can analyze the non-essential elements like genomic islands, insertion sequences, transposons and biosynthetic gene clusters according to our procedure and make iterative deletions to construct genome-minimized hosts. In our previous study, we performed similar strategy to predict two large unnecessary segments in *S. chattanoogensis* L10 genome and successfully removed them separately by Cre/*loxP* system [29]. This demonstration of feasibility lays a good foundation for constructing a minimal and more stable platform cell, which will generate a clean background for natural product discovery and a more efficient background for overproduction of value-added products.

With the rapid development of metabolomics, the genome-scale metabolic network model can also be integrated into multi-omics analysis [40]. For example, the metabolic Flux Balance Analysis (FBA) model has been used to define essential genes in silico [41–43]. Based on the minimal metabolic network analysis, we can further block the competitive and redundant metabolic pathways to enrich the fluxes into the desired pathway. In the future, the integration of comparative approaches (comparative genomics, functional genomics and pan-genomics) and experimental approaches (transcriptomics, proteomics and metabolomics) will greatly accelerate the process of constructing the hyper-performing ‘turbo cells’.

## Conclusions

In summary, the systematic analysis of *Streptomyces* genomes suggests that dispensable genomic regions take up a great proportion in the genomes. The integrative approach of comparative genomics, functional genomics and pan-genomics can not only be applied to perform a multi-tiered dissection for *Streptomyces* genomes, but also work as a universal method for systematic analysis of removable regions in other microbial hosts in order to avoid synthetic lethality of large-scale deletion and generate more miscellaneous and versatile chassis with minimized genome for drug discovery.

## Methods

### Construction of phylogenetic tree

16S ribosomal DNA (rDNA) sequences of five model representatives of *Streptomyces*, *S. albus* J1074, *S. lividan* TK24 and *S. coelicolor* A3(2), *S. avermitilis* MA-4680, *S. griseus* NBRC 13350 and other 45 *Streptomyces* genomes were selected to construct phylogenetic tree using neighbor-joining algorithm in the MEGA software. *Mycobacterium tuberculosis* H37Rv was used as outgroup. The phylogenetic tree was further visualized and edited by the EvolView online tool.

### Genome comparison and structure

The complete genome sequences of *S. albus* J1074, *S. coelicolor* A3(2), *S. avermitilis* MA-4680, *S. griseus* NBRC 13350 were obtained from NCBI (National Center for Biotechnology Information) and used for genome comparison with the progressive algorithm in Mauve 2.3.1 [44]. The match seed weight was 15 and full alignment was performed. The gap open and extend score were set to − 400 and − 30, respectively. The origin of replication (*oriC*) was analyzed by BLAST Program in DoriC [45]. The expect value was 1.0e−10 and the Matrix was BLOSUM62, and gapped alignment was performed. Other values in Mauve and DoriC were default. The genome structure was determined by calculating the deviation between *oriC* and center of chromosome.

### Proteome comparison and pan-genome analysis

The whole protein sequences of *S. albus* J1074, *S. coelicolor* A3(2), *S. avermitilis* MA-4680, *S. griseus* NBRC 13350, *S. rapamycinicus* and *S. cattleya* were also obtained from NCBI database and used for proteome comparison with OrthoVenn. The E-value was set to 1e-5 and inflation value was 1.0, and cluster relationship network was selected in OrthoVenn. The whole genome sequences of *S. albus* J1074, *S. coelicolor* A3(2), *S. avermitilis* MA-4680, *S. griseus* NBRC 13350, *S. rapamycinicus*, *S. cattleya*, *S. natalensis* and *S. clavuligerus* were used to perform pan-genome analysis with BPGA. ONE CLICK MODE was performed in BPGA program and all the analyses were performed in single step using all default parameters with identity cut off = 50% and No. of combination = 30. The KEGG/COG functional analysis was also selected. Other values in OrthoVenn and BPGA were default.

### Non-essential genetic elements and essential genes analysis

The BGCs were predicted by antiSMASH bacterial version, the GIs were analyzed by IslandViewer 4 and the ISs were determined by ISsaga2 in ISFINDER. In the antiSMASH, the detection strictness was relaxed and extra features were all on including ClusterBlast, Cluster Pfam analysis and Pfam-based GO term annotation. All values in IslandViewer 4 were default. In the ISsaga2, the number of replicon to annotate was set to 1. The essential genes and their duplications were predicted by DEG10 and ARTS. In the DEG10, the expect value of BLAST parameter was set to 1E−05. In the ARTS, the reference set was Actinobacteria and exploration mode was selected, and the HMM search mode was trusted cutoff (TC). Other parameters in antiSMASH, ISsaga2, DEG10 and ARTS were default. The circular genome map was generated by Circos [46] to visualize the distribution of genes, BGCs, GIs, ISs, and all of known essential genes with duplication were linked by Bézier curve.

### Function comparative analysis

The COG [47] and KEGG [48] database were used to analyze the function of genes. All parameters in COG and KEGG were default. The COG and KEGG distributions were sort out according to the proportion of genes in the core, accessory and unique genes.

## Supplementary information

**Additional file 1.** Determination of genome structure of *Streptomyces bingchenggensis* BCW-1. Multiple genome alignment suggested that the genome of *Streptomyces bingchenggensis* BCW-1 is asymmetric. **I***S. albus* J1074 genome; **II***S. coelicolor* A3(2) genome; **III***S. griseus* NBRC 13350 genome; **IV***Streptomyces bingchenggensis* BCW-1 genome.

## Data Availability

The datasets used and/or analysed during the current study are available from the corresponding author on reasonable request.

## Abbreviations

antiSMASH: Antibiotic and secondary metabolite analysis shell
IS: Insertion sequence
DEG: Database of Essential Genes
MGEs: Mobile genetic elements
GIs: Genome islands
BGCs: Biosynthetic gene cluster
BPGA: Bacterial Pan Genome Analysis pipeline
oriC: Origin of replication
KEGG: Kyoto Encyclopedia of Genes and Genomes
ARTS: Antibiotic Resistant Target Seeker
HGT: Horizontal Gene Transfer
COG: Clusters of Orthologous Groups
