# Supplementary figures and images for: Comprehensive dissection of dispensable genomic regions in Streptomyces based on comparative analysis approach
Source: Microb Cell Fact. 2020 May 6;19:99. doi: 10.1186/s12934-020-01359-4 (PMC7204314; doi:10.1186/s12934-020-01359-4)

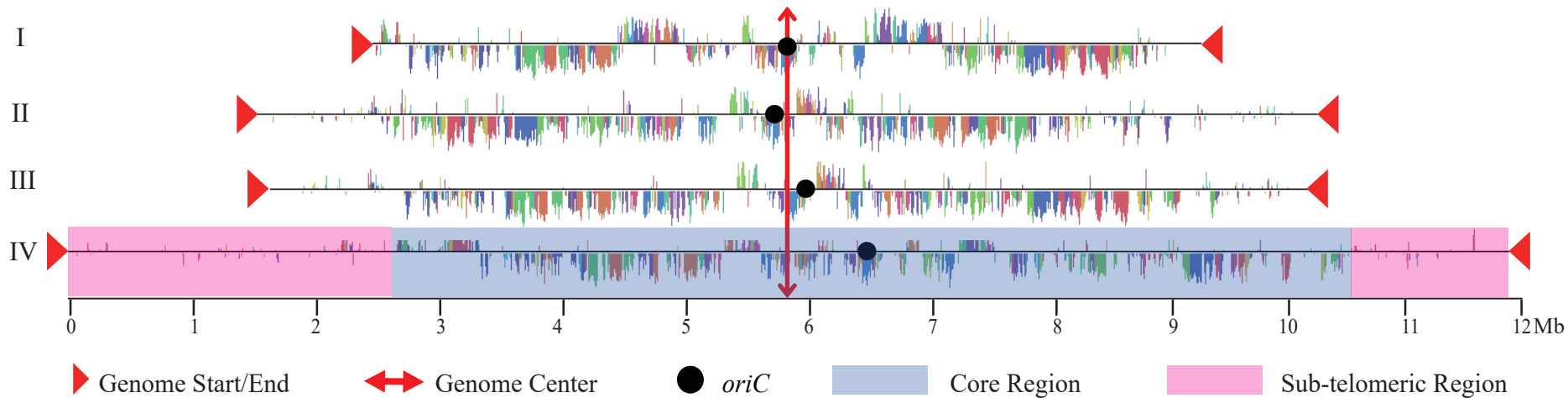

Supplement: Supplementary file 1 — Additional file 1. Determination of genome structure of Streptomyces bingchenggensis BCW-1. Multiple genome alignment suggested that the genome of Streptomyces bingchenggensis BCW-1 is asymmetric. IS. albus J1074 genome; IIS. coelicolor A3(2) genome; IIIS. griseus NBRC 13350 genome; IVStreptomyces bingchenggensis BCW-1 genome. [file 12934_2020_1359_MOESM1_ESM.pdf]
